# Occurrence and genetic variability of *Phlebotomus papatasi *in an urban area of southern Italy

**DOI:** 10.1186/1756-3305-3-77

**Published:** 2010-08-25

**Authors:** Filipe Dantas-Torres, Maria Stefania Latrofa, Domenico Otranto

**Affiliations:** 1 Dipartimento di Sanità Pubblica e Zootecnia, Università degli Studi di Bari, Valenzano, BA, Italy

## Abstract

**Background:**

A phlebotomine sand fly was noticed in the second floor of an old building in a highly urbanized area of southern Italy. A short-term entomological survey was carried out in the subsequent weeks to this event, allowing the collection of additional phlebotomine sand flies that were later identified as *Phlebotomus papatasi*. We assessed the genetic variability among *P*. *papatasi *sequences obtained in this study and those available from Italy using a mitochondrial DNA (mtDNA) fragment (from *cytochrome b *gene to NADH1) and the internal transcribed spacer 2 (ITS2) as genetic markers.

**Results:**

From 9 June to 19 July, eight males and seven females (two blood-fed) of *P*. *papatasi *were collected in the old town of Bari (southern Italy). The insects were found near the bed and in the bathroom and potential blood sources (e.g., pigeons and dogs) for them were common in the neighbourhood. Again, five females of *P*. *papatasi *collected in Valenzano, another urban area in the province of Bari, were also identified and included in the genetic study. The mtDNA sequences (945 bp) obtained from Bari and Valenzano were identical except for a single transition (T ↔ C) at the 793 nucleotide residue. Pairwise comparison of the last 440 bp of the mtDNA fragment analyzed herein with other sequences of *P. papatasi *from Italy revealed a nucleotide variation ranging from 0.2 to 1.3%. Three ITS2 sequence types were detected within specimens collected in Valenzano, one of them identical to that from Bari. Pairwise comparison of ITS2 sequences of *P. papatasi *from Italy revealed a nucleotide variation up to 1.8%.

**Conclusions:**

This study reports the occurrence of *P*. *papatasi *in an urban area of southern Italy and shows a low nucleotide difference among ITS2 and mtDNA sequences of this species available from Italy. The presence of *P*. *papatasi *in urban areas might represent a risk for human health, particularly for the potential transmission of sandfly fever viruses.

## Findings

*Phlebotomus papatasi *is the type-species of the genus *Phlebotomus*, which include all phlebotomine sand fly vectors of *Leishmania *parasites in the Old World [1]. The biology of *P*. *papatasi *has been subject of many field and laboratory investigations [2-8]. Through its range, *P*. *papatasi *is predominantly a domestic species, being often found in or nearby human habitations [9]. This sand fly feeds readily on birds, rodents, domestic animals as well as on humans [10-12]. Moreover, *P*. *papatasi *is a vector of *Leishmania major*, the agent of zoonotic cutaneous leishmaniasis in North Africa and Middle East [13], and has been implicated in the transmission of viruses in Europe [14]. Sandfly fevers were firstly described in southern Italy during the World War II, when an outbreak of influenza-like illness was recorded among US soldiers due to Sicilian and Naples viruses [15], which have been later isolated from *P*. *papatasi *in many Mediterranean countries [16,17]. The reduced abundance of *P*. *papatasi *due to the control campaigns against malaria in the past century diminished the perceived relevance of this sand fly in Italy. However, the abundance of *P*. *papatasi *is increasing [18] and the actual risk for public health in Italy is probably underestimated. Indeed, surveys carried out in Italy before 1975 indicated that *P*. *papatasi *was already widespread, thought rarely abundant [18]. However, studies conducted in central and southern Italy from 1975 onwards indicated an increase in abundance of this sand fly, especially in urban areas [18]. In spite of its widespread distribution, data on the public health significance of *P*. *papatasi *in Italy is scant and little is known about the genetic relationships among geographically isolated populations.

On 8 June 2009, a phlebotomine sand fly was noticed inside the apartment of one of us (FTD). This episode occurred on the second floor of an old, restructured building located downtown, in a highly urbanized area of southern Italy. Herein, further worth noting facts of this history are presented along with genetic data of this *P*. *papatasi *population, which is one of the southernmost records of this species in Europe. The possible public health implications of the presence of *P*. *papatasi *in urban areas of Italy are discussed.

**Figure 1:**
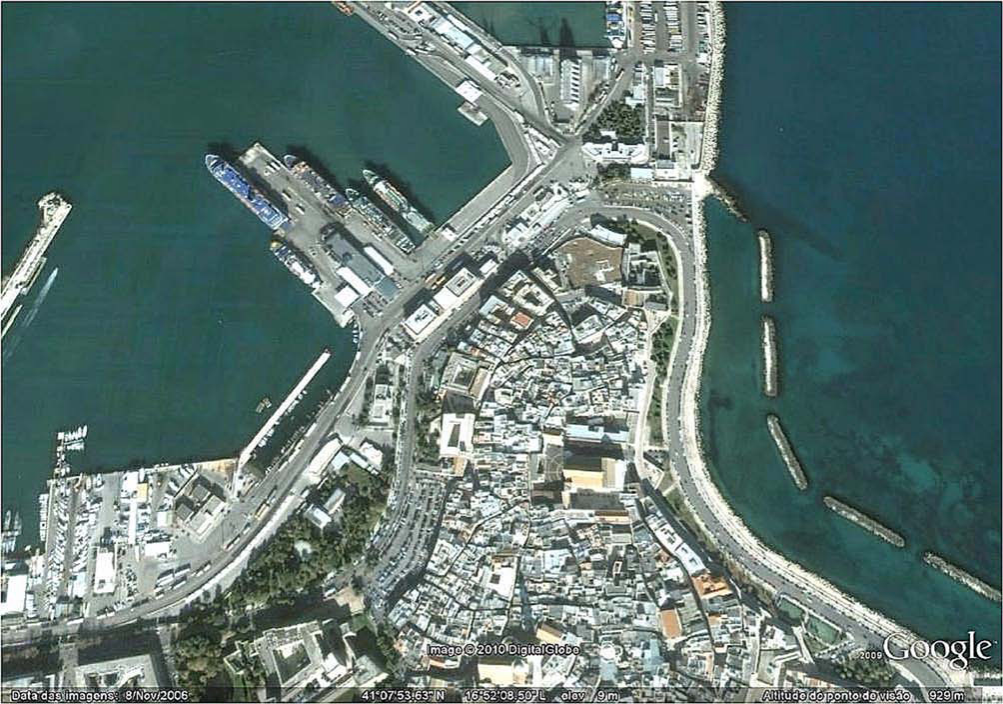
**Municipality of Bari, Apulia region, southern Italy**. Overview of the urban area where this study was conducted.

This short-term entomological survey was carried out in a building in the old part of Bari (41°07'N, 16°52'E), the main town of the Apulia region, which has a typical Mediterranean climate. The building is situated in a highly urbanized area, a few meters from the seaport of the city (Figure 1). From 9 June to 19 July, phlebotomine sand flies were collected daily using oral aspirators (every night, during 30 min, initiating around 9:00 pm), and a CDC light trap (operated daily from 8:00 pm to 7:00 am). Additionally, sticky traps were placed in two windows of the apartment and downstairs near some bushes, in a small garden belonging to the building's front yard. Phlebotomine sand flies collected were preserved in 70% ethanol until being slide-mounted in Hoyer's medium and identified based on morphology [1].

One phlebotomine sand fly from Bari was processed for DNA extraction and polymerase chain reaction (PCR) amplification of fragments of the mitochondrial - from *cytochrome b *(*cytb*) to NADH dehydrogenase subunit 1 (NADH1) - and of the ribosomal DNA - internal transcribed spacer 2 (ITS2) - as previously described [19-21]. Additionally, five specimens of *P*. *papatasi *collected from another urban area from the province of Bari (Valenzano, 41°3'N, 16°53'E) were processed for comparison purposes. In brief, DNA was extracted using DNeasy Blood & Tissue Kit (Qiagen), according to the manufacturer's instructions. The PCR was carried out in a 50 μl final volume, which included 4 μl of DNA template, 10 mM Tris-HCl (pH 8.3), 50 mM KCl, 2.5 mM MgCl_2_, 200 μM of each dNTP, 50 pmol of each primer and 1.25 U of AmpliTaq Gold (Applied Biosystems). Negative controls (no DNA) were included in each PCR run and amplicons were visualised by 2% agarose gel electrophoresis under ultraviolet exposure. PCR products were purified using Ultrafree-DA columns (Amicon; Millipore) and sequenced with an automated sequencer (ABI-PRISM 377; Applied Biosystems). DNA sequences were determined on both strands and deposited in the GenBank database (GenBank:HM992926, GenBank:HM992927, GenBank:HM992928, GenBank:HM992929, GenBank:HM992930). The electropherogram of each sequence was verified by eye, and the sequences were aligned manually using ClustalX program [22]. Sequences were compared by BLAST analysis with *cytb *and ITS2 sequences of *P*. *papatasi *available in the GenBank database. Pairwise comparison between sequences from Italy obtained in this study and those available in GenBank was carried out and nucleotide differences (D) were calculated [23].

**Figure 2:**
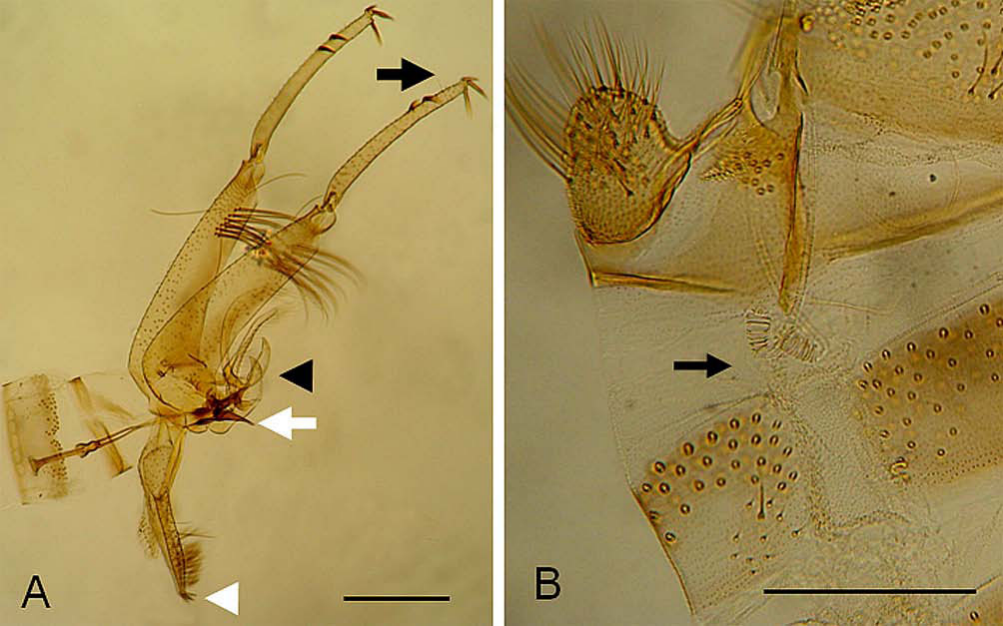
***Phlebotomus papatasi***. A: male external genitalia exhibiting style carrying five spines (three apical and two subterminal) (black arrow), the trefoil paramere (black arrowhead), tip of genital filaments (white arrow), and distal end of the lateral lobe bearing two spines (white arrowhead) (bar = 300 μm). B: female spermatheca (arrow) (bar = 100 μm).

Eight males and seven females (two blood-fed) were collected in Bari, being all identified as *P*. *papatasi *(Figures 2A and 2B). No worth noting morphological differences were noticed among the slide-mounted specimens examined in this study. All specimens were collected inside the apartment, on the second floor, being often found in the bedroom and bathroom. All attempts to collect phlebotomine sand flies with sticky traps and CDC light traps gave negative results.

The mtDNA sequences (945 bp) obtained from Bari and Valenzano were identical except for a single transition (T ↔ C) at the 793 nucleotide residue. Moreover, BLAST analysis with the longest consensus *cytb *sequence (717 bp) available in Genbank (provenience Tunisia; GenBank:AF161214), revealed a high homology (99.9%) with the sequence from Bari. Pairwise comparison of the last 440 bp of the mtDNA fragment analyzed herein with those of *P. papatasi *from Lazio (Italy) available in GenBank (GenBank:DQ381829, GenBank:DQ381830, GenBank:DQ381831) revealed a nucleotide variation ranging from 0.2 to 1.3%.

Three ITS2 sequence types were detected within specimens collected in Valenzano (i.e., nos. 1, 2 and 3), differing only by some insertions/deletions. The sequence type no. 1 was identical to that from Bari. In particular, ITS2 sequences obtained had an overall length ranging from 483 to 490 bp and presented high homologies (99-100%) with those from Syria (GenBank: DQ887666) and Cyprus (GenBank: DQ887634), respectively. Pairwise comparison of ITS2 sequences of *P. papatasi *from Italy available in GenBank (GenBank:EF408790, GenBank:EF408791) revealed a nucleotide difference up to 1.8%.

In this study, a population of *P*. *papatasi *was detected in an urban area of southern Italy, representing one of the southernmost records of this species in Europe. The morphology of both males and females of *P*. *papatasi *examined in the present study perfectly fits with the species description provided by Lewis [1]. Genetically, the specimens sequenced in this study are closely related with *P*. *papatasi *from Tunisia (*cytb*), Cyprus and Syria (ITS2). In particular, the nucleotide differences within the mtDNA sequences from Apulia herein characterized increased when compared with those from Lazio. Nonetheless, the sequence differences found in this study are consistent with those found in other studies using the same genetic markers [24,25]. These DNA fragments, particularly *cytb*, have been regarded as good genetic markers to assess the relationships between closely related populations of *P*. *papatasi *in the Mediterranean region [26]. Accordingly, our results indicate a low level of genetic variability among Italian populations of *P*. *papatasi*, which is in line with a recent phylogenetic study reporting a genetic homogeneity among 26 populations of *P*. *papatasi *from 18 countries [25].

**Figure 3:**
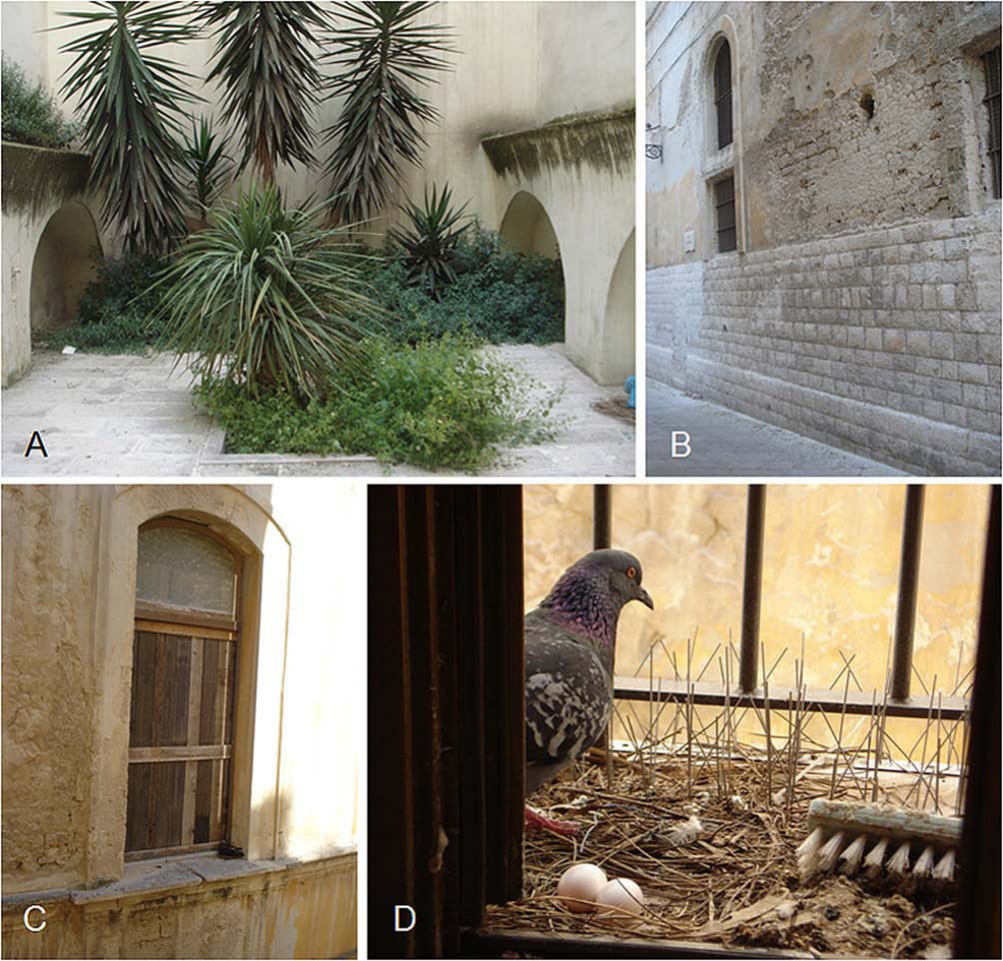
**Potential breeding sites and blood sources of *Phlebotomus papatasi***. A garden (A) belonging to the building where *P*. *papatasi *was found and the lateral wall of an old church (B) in front of the building. A closed window (C) of the same church (note a pigeon on the right bottom of the window) and a pigeon breeding in the window of the building where *P*. *papatasi *was found (D).

Based on what is known about its eco-biology [1,9], potential breeding sites of *P*. *papatasi *in this urban area of southern Italy include the earthen floor of small gardens (Figure 3A) and holes in the wall of old buildings (Figure 3B). Also, the potential bloodmeal sources could be pigeons (Figures 3C and 3D), dogs and humans. The period (June-July) in which *P*. *papatasi *was collected in this study is in line with previous records of *P*. *papatasi *in southern and central Italy, in which this species was caught from June to October [18].

Whether the presence of *P*. *papatasi *inside houses in urban areas in Italy or other southern European countries represents a risk for human health, particularly for their potential role as vectors of phleboviruses and *Leishmania *parasites, remains uncertain. Again, the natural vertebrate hosts of *L*. *major *include some wild rodents [27] whose absence in Europe might represent one of the reasons for the absence of this zoonotic agent in southern Europe [28]. Indeed, it has been stated that the risk of emergence of cutaneous leishmaniasis by *L*. *major *in Europe is low because its main gerbil reservoirs are absent, even though *P*. *papatasi *is locally abundant in some areas [28]. However, a recent study suggested that Norwegian rats (*Rattus norvegicus*), which are abundant in Europe, could be involved in the maintenance of *L*. *major *in southern Iran [29]. Indeed, the risk of introduction of *L*. *major *in southern Europe has not been fully evaluated and deserves more attention.

This work provides genetic data of a population of *P*. *papatasi *in an urban area of southern Italy, representing one of the southernmost reports of this sand fly in Europe. Finally, further research to assess whether the presence of *P*. *papatasi *in urban areas of southern Europe represents a risk for public health is needed.

## Competing interests

The authors declare that they have no competing interests.

## Authors' contributions

FDT collected and identified morphologically the phlebotomine sand flies, contributed with genetic analysis and wrote the first draft of the manuscript. MSL performed the molecular characterization and genetic analysis of *P*. *papatasi*. DO contributed with genetic analysis, interpretation and revision of the manuscript.
